# A randomized trial of the Caregiver Interaction Profile (CIP) training with childcare providers: the Copenhagen Daycare Project study protocol

**DOI:** 10.1186/s40359-024-01568-1

**Published:** 2024-03-06

**Authors:** Sophie Reijman, Clara Christensen Vieira, Tina Wahl Haase, Katrien O. W. Helmerhorst, Maiken Pontoppidan, Sofie Amalie Grosen, Ida Egmose, Katrine Røhder, Mette Skovgaard Vaever

**Affiliations:** 1 Center for Early Intervention and Family Studies, University of Copenhagen, Copenhagen, Denmark; 2 Department of Pedagogy and Educational Sciences, Child and Family Welfare, University of Groningen, Groningen, The Netherlands; 3 Danish National Center for Social Science Research, Copenhagen, Denmark

**Keywords:** Child care, Interactive skills, Training, Randomized controlled trial

## Abstract

**Background:**

Most young children (0–3 years) attend formal childcare in Denmark, many of them fulltime. Yet recent reports of the quality of Danish childcare centers have shown that in more than one-third of nurseries, the interactions between caregivers and young children (0–3 years) are of “insufficient” quality, which constitutes a risk for affected children’s well-being and development. Effective interventions to improve childcare providers’ interactive skills are necessary.

**Methods:**

In this randomized controlled trial, we test the effectiveness of the Caregiver Interaction Profile training, which focuses on improving six core interactive skills: sensitive responsiveness, respecting children’s autonomy, structuring and limit setting, verbal communication, developmental stimulation, and fostering positive peer interactions. We will recruit *N* = 200 childcare providers from nursery groups in Copenhagen (*n* = 100 training group, *n* = 100 waiting-list control group). Our primary outcomes are childcare providers’ six interactive skills named above, observed from video-recorded interactions in the nursery groups. The secondary goal of our study is to test whether the training boosts children’s social-emotional and linguistic development. To this end we aim to recruit *N* ≈ 500 children from participating childcare providers’ nursery groups (*n* ≈ 250 training group, *n* ≈ 250 waiting-list control group). We measure social-emotional and linguistic development with various standardized questionnaires, filled out by parents and childcare providers.

**Discussion:**

If the training is effective at improving childcare providers’ interactive skills, then this will be an important foundation for implementation efforts, such as offering the training as part of the educational program of childcare providers. Future research should also evaluate whether the Caregiver Interaction Profile training is effective for childcare providers of older children (3–5 years) in Danish kindergartens.

**Trial registration:**

This trial is registered at clinicaltrials.gov as “Testing the Effects of the Caregiver Interaction Profile Training on the Interactive Skills of Daycare Providers (CDP)” with registry ID NCT05654116. Registration date: 12/01/2022.

## Background

For children attending formal childcare, the quality of care they experience affects the course of their development [e.g., 1]. In Denmark (where the current study takes place), more than half of 0-2-year-olds and almost all 3-5-year-olds attend childcare at least 30 h a week [2], which makes childcare providers (CPs) essential in the early relationships that shape children’s lives. Research into childcare quality distinguishes between “structural quality” (characteristics of the childcare center such as group size and the educational background of staff) and “process quality” (dynamic features such as the interactions between caregivers and children) [3]. A literature review has suggested that structural features affect child development indirectly via process quality; for instance, with more staff available in relation to the number of children, CPs’ capacity to give emotionally supportive care improves [4]. Research has consistently shown that children who receive sensitive and stimulating care in childcare, show better social-emotional, cognitive, and language development than children who experience lower-quality interactions [e.g., 1, 5, 6, 7]. While there is less knowledge on process quality in child-care centers for the youngest children (0–2 years) [4], a recent national study by the Danish National Center for Social Science Research (VIVE) and the Danish Evaluation Institute (EVA) showed that in more than a third of Danish nurseries where children between 6 months and 3 years receive childcare, the quality of the interactions between childcare providers and children received an “insufficient” qualification [8]. This shows that improvement of interactive behaviors of CPs in childcare centers is needed to support infants and toddlers in their development. In the current paper, we describe a study in which we test the effectiveness of the Caregiver Interaction Profile (CIP) training. This training program is specifically aimed at improving CPs´ interactive skills with the children in their care, and which has shown good effectiveness in a Dutch randomized controlled trial [9]). In the present study, we test whether the CIP training improves CPs´ interactive skills in Danish nurseries, which infants and toddlers attend (6 months– 3 years). Although the CIP training does not directly target child development, if the training leads to a more supportive and stimulating caregiving environment, then we would, based on developmental theory and research, predict that child development will be positively affected. We therefore evaluate whether the CIP training also leads to improved social-emotional and language development in children.

### Development of the CIP scales and training

The CIP training is based on the CIP scales, an observational measurement of the following interactive skills in CPs: (1) *sensitive responsiveness*, which refers to CPs’ prompt and adequate responses to children’s signals and needs, (2) *respect for children´s autonomy*, which is demonstrated when CPs explicitly validate children’s intentions and ideas, and encourage them to try things out for themselves, (3) *structuring and limit setting*, which is a CP’s ability to clearly communicate expectations toward children and set clear and consistent limits, (4) *verbal communication*, which refers to the frequency and quality of verbal caregiver-child interactions, (5) *developmental stimulation*, i.e., deliberate attempts by the CP to foster children´s motor skills, cognitive development and/or creativity, (6) *fostering positive peer interactions*, which reflects the degree to which a CP facilitates, encourages, and stimulates positive interactions between children [10]. The CIP scales were based on the following process: Based on their systematic review of theoretical frameworks and empirical research in developmental science, the authors identified these six interactive skills as being important contributors to high-quality interactions between caregivers and 0–4 year-old children in childcare. Next, the researchers surveyed four groups of stakeholders in childcare (parents, CPs, childcare center directors, and external experts) who rated the perceived importance of the six caregiving skills along with other indicators of process quality in childcare: the six skills were perceived as important or very important by all groups of stakeholders, with high levels of agreement [10]. Finally, a review of observational instruments showed that no existing instrument measured these six caregiving skills at the group level in childcare, consequently the researchers developed and validated the CIP scales [10]. Scales 1–3 (see above) were adapted from existing scales developed by De Schipper and colleagues (De Schipper & De Riksen-Walraven, 2004, unpublished manuscript, see [11]), while scale 5 (developmental stimulation) was adapted from a scale in the Observational Record of the Caregiving Environment (ORCE; [12]), and scales 4 (verbal communication) and 6 (fostering positive peer interactions) were created by Helmerhorst and colleagues [10; see also^ 9^]. A national study on process quality in 200 randomly selected childcare centers in the Netherlands using the CIP scales showed that on average, CPs had adequate-to-good scores on sensitivity, respect for autonomy, and structuring and limit setting, but lower scores on verbal communication, developmental stimulation, and fostering positive peer interactions, with particularly inadequate scores on the latter two scales [13]. The authors concluded that this was consistent with international research findings that CPs generally score higher on fundamental caregiving skills, like sensitivity, than on educational skills like developmental stimulation, and they highlighted the need for professional training of CPs in these interactive skills. The authors went on to develop the CIP training, which focuses on improving the six interactive skills in CPs [9]. Through a review of meta-analyses on interventions in childcare [14, 15], they found no existing training program that addressed these skills together. They also identified the following components as characteristic of effective training programs: (a) individual (rather than group-based) sessions, (b) the use of personalized video-feedback, and (c) shorter trainings (< 10 h) were found to be just as effective as longer trainings (> 10 h) [15, see 9, for a more detailed description of the development of the CIP training]. Based on these findings, the CIP training was developed. The CIP training is a relatively brief (5 × 2 h), individual training using personalized video-feedback.

### Theoretical foundations and assumptions of the CIP scales

Focus on the six CIP skills for quality measurement and training is rooted in developmental theory and research. The developers of the CIP scales cite attachment theory [16, 17] and Erikson’s stages of development [18] as theoretical fundaments for sensitivity and respect for autonomy, respectively. A basic tenet of attachment theory is that infants are predisposed to form an attachment relationship with those who consistently provide in their caregiving [17], including CPs [19]. Children’s attachment behavioral system becomes activated when they feel vulnerable, for example when they are upset, scared, tired, or ill. In those moments, the set goal of the attachment behavioral system becomes *felt security*, which can be obtained through nearness to or contact with an attachment figure (this function of the attachment figure has been referred to as the “safe haven”; [20]). In those moments, through repeated experience, infants learn whether their bids for comfort are met with sensitivity, i.e. prompt and appropriate responses. Once felt security has been achieved, the attachment behavioral system deactivates and children are again motivated to explore the environment, using the availability of the attachment figure as a “secure base” from which to do so [16]. Sensitive responsiveness is therefore crucial in an attachment figure’s capacity to be both “safe haven” and “secure base” for the children in their care.

The importance for caregivers to show *respect for children’s autonomy* can be traced back to Erikson who emphasized autonomy (conceptualized as a sense of control and self-direction) as a crucial developmental task for toddlers [18]. It is also an intricate part of attachment theory: “Cooperation (versus interference) with ongoing behavior” was the second scale of Ainsworth’s Maternal Caregiving and Interaction Scales and thought to be a prerequisite for the caregiver’s position as a “secure base” [16]. The opposite of respecting children’s autonomy is intrusiveness, which can manifest, for example, as abruptly interrupting children in their activities, or overruling their ideas and perspectives with one’s own. Markedly intrusive care can have negative effects, even for infants [21, 22].

A combination of high levels of sensitivity and respect for autonomy as well as *structuring and limit setting*, which refers to communicating expectations clearly to children and setting boundaries in a clear and consistent way, matches the profile of an authoritative caregiving style as identified by Baumrind [23], which has predicted long-term positive adjustment in children [e.g., 24].

While the developers of the CIP scales did not base the scales *verbal communication* or *developmental stimulation* directly on Vygotsky´s [25] concept of the zone for proximal development, we acknowledge here there are similarities between Vygotsky´s concept and the assumptions about learning implicit in the scales. The zone for proximal development refers, broadly speaking, to the difference between what children are able to achieve on their own and what they are able to achieve with the guidance of a capable other (adult or peer): the “zone” represents their proximal developmental potential. When CPs talk with children and connect to children´s interests and appropriately challenge their capacities, offering supportive guidance in language and activities, they capitalize on children´s learning potential. Research has shown that children who experienced more speech directed at them, had a larger expressive vocabulary [26, 27]. Also, more developmental stimulation by CPs uniquely predicted increased cognitive development in infants, even when controlling for caregiver sensitivity and children´s earlier cognitive development [6, 28].

The sixth interactive skill, *fostering positive peer interactions*, recognizes that children do not only interact with CPs but are also part of a community of peers in childcare. CPs can praise kind interactions between peers, thereby stimulating them (in part) through positive reinforcement. While there is little research on peer relationships for infants and toddlers, one study has suggested that negative peer interactions at 15 months predicted aggressive behaviors (as reported by CPs) at 23 months, while positive peer interactions at 15 months predicted children´s (CP-reported) wellbeing in childcare at 23 months [29]. The study also suggested that at younger ages, children may need particular guidance from CPs in interacting with peers, as negative initiatives toward peers increased over time while positive responses to initiatives from peers decreased [29]. Positive experiences of preschoolers with their peers in kindergarten can even buffer negative effects of negative caregiving experienced at home [30].

### Cross-cultural considerations

We are aware that evaluating CPs on these skills and assessing children’s development as an outcome of the CIP training explicitly involves making normative judgments about what we consider positive developmental outcomes for children, and what are better and worse ways of caregiving in order to achieve those developmental outcomes. The vast majority of developmental science, including the research we cite above, is based on Western, White, urban, middle-class samples [31, 32]. We do not presume that the values ingrained in the interactive practices of the CIP training are universal. For instance, while research has indicated that sensitivity is considered an important caregiving quality across cultures, this was less so in rural, large, low-income families, and some cultural differences were found as well [33]. We note that anthropological research has reported on, for instance, the Runa, an Indigenous people in the Ecuadorian Amazon, whose parental practice is to not accede to a young child´s will and bids for attention (while otherwise rejoicing in the children; [34]). Regarding verbal communication, cultural differences have also been found in parental practices of child-directed talk [35]. In short, the selection of our outcome measures is culturally conditioned.

The CIP training was first developed and tested in the Netherlands, where the six caregiving skills were considered relevant indices of childcare quality by different groups of stakeholders (e.g., parents, CPs; [10]). For the current study we have not done a cultural validity assessment, because the Netherlands and Denmark are both WEIRD (Western, educated, industrialized, rich, democratic) countries, and the daycare contexts in both countries share common features (e.g., the majority of Dutch and Danish CPs have received a broad pedagogical training). The CIP scales have also been used in Norway [36, 37]. Recently, different stakeholders in Bangladesh, including CPs and parents, rated the CIP skills as important-to-very important elements of process quality in childcare [38]. Continued research on CIP´s cross-cultural applicability will be needed.

### The present study

In this randomized controlled trial, we test the following research questions:

1. Does the CIP training enhance the interactive skills of Danish CPs? Based on previous research [9], we hypothesize that CPs who receive the CIP training will improve significantly on the six interactive skills.
2. Does the CIP training enhance the social-emotional and linguistic development of children who are in the care of trained CPs? The effects of the CIP training on the children on childcare has not been evaluated previously, but based on a meta-analysis showing an overall small effect of targeted interventions in childcare on children’s social-emotional development [16], we tentatively expect to find a small positive effect of the CIP training on children’s social-emotional development. Another meta-analysis has shown that when CPs participate in personal development courses or coaching in relation to language, this may have a small or small-to-moderate effect on children’s language development, depending on the measured outcome (small effects for receptive vocabulary and alphabet knowledge, a small-to-moderate effect on phonological awareness; [39]. We therefore tentatively expect small effects of the CIP training on children’s linguistic development.

## Method

### Study setting

The Copenhagen Daycare Project is a collaboration between the University of Copenhagen, the City of Copenhagen, University College of Copenhagen, and Groningen University.

### Participants, recruitment, and allocation

We aim to recruit 200 childcare providers (*n* = 100 training group, *n* = 100 waiting-list control group) in nursery groups (attended by children aged 6 months-3 years) in the City of Copenhagen. We also aim to recruit *N* ≈ 500 children in the care of participating childcare providers (*n* ≈ 250 training group, *n* ≈ 250 waiting-list control group). All childcare providers (CPs), with or without formal training, can take part in the study, except for explicitly temporary staff, due to high risk of drop-out. All children can participate as well, with the consent of their parents; only children of whom it is known they will not continue in the same care group or childcare center for the duration of data collection are not enrolled.

Recruitment started in spring 2022. The City of Copenhagen on a regular basis sends out an advertisement for the research project as part of a newsletter to all childcare centers in Copenhagen. Interested center leaders are invited to send their details to a designated contact person at the City of Copenhagen, who forwards the details to the research team. The CDP research team contacts center leaders to tell them more about the study, answer any questions, and schedule an in-person introductory meeting with staff at the childcare center. After the introductory meeting, the center leader contacts the research team with the names of staff who will be participating. Childcare centers consist of multiple “care groups”, or nurseries, i.e. rooms attended by a group of infants/toddlers (often 9–12) and 3–4 CPs (according to Danish law, each CP can care for a maximum of 3 children under 3 years old). We randomize at the level of the care group, because we cannot test CIP training effects on children if within a care group, some staff were assigned to the training group and other staff to the control group. In order to avoid large clusters, a maximum of 2 staff members from each care group can participate. Therefore, if a childcare center participates with multiple care groups, there will be groups allocated to the training group and to the control group within the same center. We explicitly request participants in the training group not to share training content with their colleagues from different care groups, in order to minimize a treatment diffusion effect (i.e. interactive skills in the control group improving due to exposure to training material).

For children in Copenhagen, the municipality administration on a yearly basis calculates their “economic social cultural status” (ESCS) index, based on parents´ educational background, income, and occupation. Index values typically range from − 1 to + 1, with 0 representing the average in the municipality of Copenhagen; children’s index value can therefore only be interpreted comparatively (i.e., the same educational background, income, and occupation could get a higher or lower value from one year to the next, depending on shifts in the Copenhagen average). In our recruitment, we aim for representative variation in the ESCS indices of the children at child-care centers. Therefore, the City of Copenhagen provides us with the average ESCS index of the children at each enrolled child-care center. Based on these, recruitment may be targeted more toward child-care centers at different index levels.

Additionally, the municipality of Copenhagen conducts yearly evaluations of the quality at childcare centers in Copenhagen within multiple domains, including “social relations– contact between adults and children”, “inclusion and child community”, and “language”. For each of these domains, childcare centers get a rating of “no recommendations needed” (the highest rating), “adjustments recommended”, or “new efforts required” (the lowest rating). The yearly reports are publicly available. As with the ESCS index, we aim for variation in the quality evaluations in our sample, and particularly aim to include childcare centers with at least one recommendation of “adjustments recommended” or “new efforts required”, to mitigate the risk of a ceiling effect whereby already highly competent CPs may experience limited benefits from the CIP training.

### Procedures

**Fig. 1:**
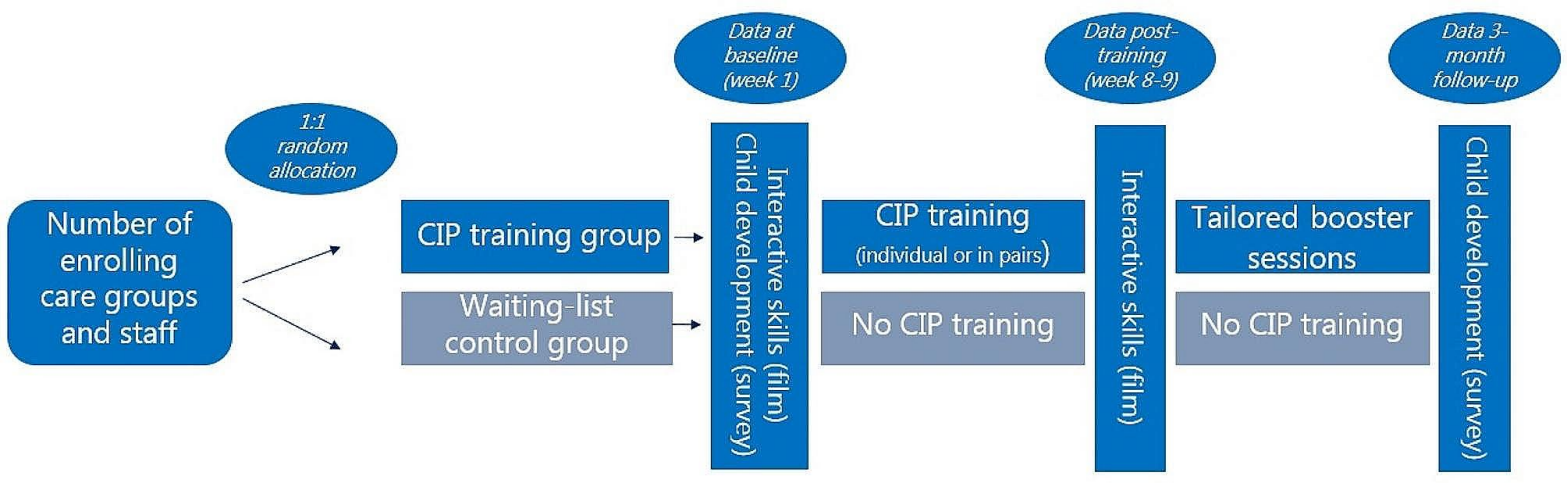
Schematic overview of random allocation and data collection

For a schematic overview of post-enrolment procedures, see Fig. 1. After enrolment, one of the study coordinators sends anonymized data to an external researcher (MP) who randomizes the care groups to the training or waiting-list condition with a 1:1 ratio. Following random allocation, study coordinators send out consent forms and information sheets for the CPs at the childcare centers and for parents of the children in the relevant care groups. The center leader and participating CPs distribute information sheets to parents and with permission pass on parents’ contact details to the research team. Parents receive a link to a digital consent form and questionnaires about their children’s development on the Danish digital platform SurveyXact. Additionally, center leaders and CPs collect written parental consent with paper copies of consent forms. Parents can separately choose to participate or not in (1) their child being part of video recordings made to assess CPs’ interactive skills (see Figs. 1), (2) answering questionnaires on their children’s development, and (3) allowing CPs to answer questionnaires on their children’s development. If parents do not consent to their child being in video recordings, we avoid filming the child. Baseline measurements of the six interactive skills in the training and waiting-list nurseries are completed through video recordings by student assistants. Additionally, parents and CPs of children in training and waiting-list nurseries fill out the Ages and Stages Questionnaire: Social-Emotional, Second Edition (ASQ:SE-2; [40; 41]), the Strengths and Difficulties Questionnaire (SDQ; [42]), the Child Behavior Checklist (CBCL; [43]), and the brief Communicative Development Inventories for childcare (CDI:Edu; [44]) as a baseline assessment of children’s social-emotional and language development. One-to-two weeks later, CPs in the training group start their CIP training individually or in pairs (with trainings lasting 5 or 6 weeks, respectively; see Table 1). Two weeks after completion of the CIP training, CP-child interactions in both the training and waiting-list nurseries are filmed again by student assistants. We aim for students assistants to be blind to the training versus control condition as much as logistically possible. Three months later, parents and CPs fill out the ASQ:SE-2, the SDQ, the CBCL, and the CDI to measure potential intervention effects on children’s development. In the space of those three months, CPs in the training condition are offered a 2-hour booster session per person; they can also choose to have a 1-hour individualized booster sessions and, after the 3-month lapse, to have a staff meeting at which key points from the CIP training are presented to everyone at the childcare center (using video modelling but not individualized feedback).

### CIP training group

CPs in 50% of the enrolled daycare groups (randomly allocated) will receive the CIP training group, starting one-to-two weeks after baseline assessments. Participants do the training either individually or in pairs. The structure for each training format is shown in Table 1.

**Table 1:** Structure of CIP training delivered to individuals or pairs

	Session 1	Session 2	Session 3	Session 4	Session 5	Session 6
**Individual**	SR and RA	SL and VC	DS and FPPI	Recap of two skills CP can choose	Session shared with a colleague	*Not applicable*
	*2 hours*	*2 hours*	*2 hours*	*2 hours*	*2 hours*	
**Pairwise**	SR	RA	SL	VC and DS	FPPI	Recap of two skills CPs can choose together
	*2 hours*	*2 hours*	*2 hours*	*3 hours*	*2 hours*	*2 hours*

Note: SR = sensitive responsiveness; RA = respect for autonomy; SL = structuring and limit setting; VC = verbal communication; DS = developmental stimulation; FPPI = fostering positive peer interactions; CP = childcare provider

Each session has a standardized set-up. For the skill in focus in any week, participants first read a description of the skill in the CIP manual. After they have become familiar with the skill at a descriptive level, they watch standardized example videos of the interactive skill at a high, medium, and low level. The CP and the CIP trainer discuss why the level of the interactive skill in the video clip is high/medium/low. Subsequently, CPs watch pre-selected video clips of themselves in interactions with children in their care. The CIP trainer asks the CP to reflect on what they see in the footage in relation to the relevant skill. Through questions, the CIP trainer guides the CP to identify what goes well in their interactions, and if relevant, what they might do even more, or would do differently based on what they see. At the end of the session, the CP(s) marks on a checklist or writes down what goes well and what they would like their working points to be (i.e., concrete behaviors to focus on while they work during the week and discuss again during the next session). The design of the training program (e.g., the video footage shown, the nature of the CIP trainer´s questions) is purposefully constructive and non-judgmental, with an emphasis on the interactive strengths of the CP. In the sessions devoted to a recap of two skills selected by the CP(s), the skills are discussed again and new personalized video material is shown. In CIP trainings delivered to individuals, the 5th session can be shared with a colleague who also follows the CIP training individually, and colleagues can show each other clips from their training which they selected. The CIP training is described in detail by Helmerhorst and colleagues [9]. In the pairwise training program, most sessions focus on one skill each (as opposed to two skills), because otherwise we estimated the number of shown video clips per session would be too high for effective learning; in order to limit the number of sessions we make an exception for the skills Verbal Communication and Developmental Stimulation, which we combine together in a 3-hour session (see Table 1).

CIP trainers SR, CCV, TWH as well as Dr Vibe Larsen (University College Copenhagen) were trained in the CIP protocol by the original CIP trainer and fourth author, KOWH. Later in the project, three additional CIP trainers were trained by authors CCV and SR. Additional CIP trainers may be trained by author KR. CIP trainers convene monthly for CIP training trouble-shooting and fidelity meetings, led by SR or KR, in which we score interactive skills together, develop suitable feedback based on selected video fragments, and discuss potential doubts or questions around specific trainings, among other things.

### Waiting-list control group

CPs who were randomly assigned to the control group will take part in the same assessments as the training group (see Fig. 1), but they will not receive any CIP training in between. At baseline and follow-up we ask CPs if they have participated in any other skill development programs, and which one(s). After the last assessments of the research protocol are finished, participants in the control group will also receive the CIP training, either individually or pairwise, as they prefer.

### Primary outcome measures

*The Caregiver Interaction Profile (CIP) Scales* were developed by Helmerhorst et al. [10] as an observational method to assess caregiving quality in the daycare setting, focusing on the interactive quality between CPs and children aged 0–4 years. The CIP scales focus on six interactive skills of daycare providers: *(1) Sensitive Responsiveness*, that is, the degree to which the daycare provider responds promptly and appropriately to children’s signals, *(2) Respect for Children’s Autonomy*, which refers to the degree to which the daycare provider encourages children to try things out for themselves or in their own way, and values their perspectives and intentions, *(3) Structuring and Limit Setting*, or the degree to which the daycare provider structures activities so all children may benefit, and clearly communicates to children what is expected of them, *(4) Verbal Communication*, which reflects the frequency and quality of the verbal interactions between the daycare provider and the children, *(5) Developmental Stimulation*, or the degree to which the daycare provider stimulates children’s development, such as cognitive or motor development or creativity, and *(6) Fostering Positive Peer Interactions*, that is, the degree to which the daycare provider recognizes positive interactions between children and actively promotes them (e.g., taking turns, sharing, playing together). Each skill is scored on a scale from 1 to 7 (1 = very low, 2 = low, 3 = moderately low, 4 = moderate, 5 = moderately high, 6 = high, 7 = very high). The CIP scales have been shown to be reliable and valid for use in childcare centers for 0- to 4-year-old children in The Netherlands [10, 45].

The six CIP skills are coded based on video-recorded interactions in four different situations during the day (with no specific instructions for the CPs): free play, lunch/snack, diaper change, and a transition between group activities (recorded for 10 min each). CIP scores are given for each video recording separately, and are then averaged across the four situations for the purpose of data analysis.

All but two coders were trained by fourth author KH and Dr Gevers Deynoot (both members of the Netherlands Consortium for Research in Child Care, which developed the CIP scales); two subsequent coders (at the moment of writing this) have been trained by the first author, SR. Future coders may be trained by the 7th author, IE. All coders were thoroughly trained, and after training coded a reliability set of 12 videos: coders had to meet at least 80% agreement within a 1-scale point range with the scores given by expert coder KH. Trained coders check their inter-rater agreement on a regular basis during the entire coding process. For every 20 videos (of 5 CPs in 4 different situations), 4 videos (of different CPs in different situations) are double-coded by “reference coder” IE. Thus far, agreement (i.e. being within 1-scale point range) between the coder and reference coder IE is always at least 80%. In cases of disagreement (more than a 1-scale point difference), videos are re-watched where needed and the coders find a consensus through discussion. All coders are blind to the allocation of the CPs they code.

### Secondary outcome measures

We will examine the effect of the CIP training on children’s social-emotional development, using the Ages and Stages Questionnaire: Social Emotional-version 2 (ASQ:SE-2; [41]), the Strengths and Difficulties Questionnaire (SDQ; [42]), and the Child Behavior Checklist (CBCL 1,5–5; [43]), filled out by parents and CPs. Other translations are available if parents are not fluent in Danish. We selected these three questionnaires on social-emotional development because a goal of our project outside of the CIP trial is to compare children´s scores on the three measures to assess convergent validity, given the need for validation research on these widely used measures in the Danish context. Because *verbal communication* is a skill that is part of the CIP training, we will also evaluate training effects on children’s language development, using the Danish Communicative Development Inventory short form for Educators (CDI-Edu; [44]).

The *Ages and Stages Questionnaire: Social Emotional-version 2* (ASQ:SE-2) is an instrument to screen infants and young children for potential risk or delay in seven dimensions of social-emotional development, based on observed behaviors: self-regulation, compliance, communication, adaptive functioning, autonomy, affect, and interactions with people [40, 41]. Different versions of the questionnaire exist for different age groups; we used the versions for 6 months (23 items), 12 months (27 items), 18 (31 items), 24 (31 items), 30 (33 items), and 36 months (35 items) depending on the age of the participating children. Answer options for respondents are “*rarely/never*” (0 points), “*sometimes*” (5 points) and “*often/always*” (10 points). In addition, respondents can indicate whether they feel concerned about the child in relation to any of the items by checking a box; a checked box adds 5 points for that item. Therefore, the maximum score per item is 15. The total score is calculated by adding up the item scores, and a higher score indicates more developmental risk/delay.

The ASQ:SE-2 is a widely used research tool to assess potential developmental concerns [46] and has been translated to multiple languages. Systematic reviews of its psychometric properties have shown good internal consistency (whether various items measure elements of the same construct), test-retest reliability (whether repeated assessments yield similar outcomes), sensitivity (whether it detects development problems when these are present), and specificity (whether it does *not* signal developmental problems when these are absent) [46, 47]. For sensitivity and specificity assessments, the Child Behavior Checklist (CBCL; [43]) has mainly been used as a comparison instrument. Preliminary psychometric data on the Danish translation of the ASQ:SE-2 suggest that the internal consistency of items is acceptable (Cronbach’s α range 0.70–0.79) for the 12-, 24-, and 36-months questionnaires (Pontoppidan, 2019, unpublished presentation).

The *Strengths and Difficulties Questionnaire* (SDQ; [42]) is a brief, well-validated, widely used questionnaire about children’s social-emotional behaviors. It consists of 25 items with answer options “not true” (0 points), “somewhat true” (1 point) and “certainly true” (2 points). In this study, we use a Danish translation of the version for 2-4-year-olds. It contains items like “*is generally liked by other children*” and “*mostly does what he/she is told*”. The items are divided into 5 subscales measuring emotional symptoms, conduct problems, hyperactivity/inattention, peer relationships problems, and prosocial behavior. Scores for the first four subscales are summed to get a total difficulty score (range 0–40), and the prosocial items are summed to get a total prosocial score (range 0–10). While there are no psychometric data available for the Danish version of the 2-4-year questionnaire, a psychometric study in Denmark with 5-12-year-olds and Swedish research with 2-4-year-olds support its valid and reliable use [48, 49]. Because there are no versions of the SDQ for children below 2 years of age, we only asked parents and CPs of children aged 2 or older to fill out the SDQ.

The *Child Behavior Checklist 1.5-5* (CBCL; [43]) measures social-emotional and behavior problems in infants and young children. It contains 99 brief items describing different behaviors considered potentially problematic for children’s development, including fear to try new things, medically unexplained pain, and defiance, and caregivers indicate on a scale of 0–2 whether these are “*not true*”, “*somewhat/sometimes true*”, and “*very/often true*”. Scores for the 99 items are summed to reach a total problem behavior score. Higher scores therefore in principle reflect more behavioral problems, although somewhat elevated scores still fall within the normative, expected range for infants, toddlers and preschoolers (the average score in a Danish general population sample was 17.3; [50]. It is a validated and widely used instrument: for example, it was able to identify preschoolers with different disorders, particularly externalizing symptoms [51]. The CBCL 1,5–5 has been used for diagnostic purposes in Danish clinical research [52].

The *CDI:Edu* [44] is a Danish short form of the Communicative Development Inventories (CDI; [53], adapted for the childcare setting by replacing vocabulary items unlikely to be used in childcare. The original MacArthur-Bates CDI has been found a valid instrument for measuring receptive and expressive language development from late infancy to toddlerhood [54], and the same holds for its Danish adaptation [55]. The CDI:Edu measures development of the Danish language in children aged 18–36 months and contains 70 vocabulary items; respondents mark words if the child a) understands them, or b) understands and says them. The total number of words marked is the total vocabulary score. It takes about 10 min to complete for one child, which CPs can do in a valid and reliable way: for instance, CDI:Edu scores correlated as expected with gender (girls scoring higher) and maternal educational background (more advanced language development when mothers had more years of formal education), and the items had good internal consistency [44]. In our study, both CPs and parents complete the CDI:Edu. While there is also a Danish short form of the CDI [56], this was developed as a screening tool of language delays in older toddlers, while the CDI:Edu can be used for toddlers of a wider age range.

It is worth noting here that in Denmark, childcare is monolingual: children are exposed only to the Danish language. For children with a different native language (or multiple languages at home), the development of their native language and their secondary/tertiary language (i.e., Danish) may be interconnected, but study findings have suggested this may be a one-way street: stimulation of children’s native language(s) can positively affect learning of their secondary/tertiary language, but not the other way around [57]. We therefore hypothesized that the CIP training would stimulate children’s Danish language development, but would not necessarily affect their vocabulary in another language. We therefore only measure children’s development of the Danish language with the CDI:Edu.

### Power analysis

The current study is a 3-level clustered randomized trial (CRT): CPs (level 1) are nested within care groups (level 2), which are nested within childcare centers (level 3). The multilevel nature of CRTs requires special consideration when doing power calculations. In order to detect main effects of the CIP training, maximizing the number of units at level 2 and especially 3 will benefit statistical power more than maximizing the number of individuals at level 1 [58]. In order to detect effects of level 2- or 3-moderators (for instance, ESCS index of the childcare center, a level 3-moderator), CRTs generally need many more clusters than for main effects, and moderating effects are typically smaller than main effects, so that testing for level 2- or 3-moderators is often financially and practically unfeasible [58]. However, in order to detect moderators at level 1, maximizing the number of individuals within clusters benefits power most [58]. We have determined our sample size at level 1, 2 and 3 by calculating the minimally detectable effect size (MDES) for main effects of the CIP training on CPs´ interactive skills and children´s social-emotional and linguistic development. In these considerations we also had to strike a balance between investing resources toward training CPs in as many childcare centers (units at level 3) as possible to achieve high statistical power for analyses on our primary outcome measures (CPs’ interactive skills), while also including enough CPs within each care group (level 1) so that the children in the group might benefit more from their CPs´ training, leading to larger training effects on child development.

In order to balance our investment of resources on maximizing training effects on CPs´ interactive skills (by striving for recruitment of CPs from as many childcare centers as possible) and on maximizing training effects on child development (by training as many CPs in each care group), we decided to include a maximum of 2 CPs per care group. Some center leaders only want 1 CP per care group to participate, presumably due to difficulties arranging for alternative staff during training hours (despite the City of Copenhagen financing alternative care). Therefore, per care group, 1–2 CPs participate in the study. Through power calculation in PowerUp! (see https://www.causalevaluation.org/power-analysis.html; [59]), we estimated that with an average of 2 CPs per care group, 3 care groups per childcare center, and a total of 35 childcare centers, we will have a power of 0.84 at (*α* = 0.05) to detect an MDES of 0.35, which was the average ES in a meta-analysis of RCTs testing different trainings on CPs’ interactive quality [15]. In the first RCT of the CIP [9], which came out after the above meta-analysis, the ES for *structuring & limit setting* was also 0.35, while the other effects were larger: 0.59–0.79.

Because childcare interventions focused on improving process quality do not directly target child outcomes, the effects of these interventions on children tend to be smaller (overall ES of 0.26 in [15]). We estimated that with an average of 5 children per care group, 3 care groups per childcare center, and a total of 35 childcare centers, we will have a power of 0.80 to detect an ES of 0.26.

### Data analysis

A consequence of the nested structure of our data is that due to similarities within care groups and institutions (e.g. CPs in the group interact with the same children, and may have shared strategies for implementing principles from the CIP training), observations and assessments with different CPs from the same care group or institution are unlikely to be independent from each other. In order to account for this, we will use multilevel modelling to test the effect of the CIP training on CPs’ interactive behavior and on children’s social-emotional and linguistic development. We will test the effect of the CIP training (versus control group) on the six CIP skills using linear regression analyses, adjusting for baseline levels of the CIP skills. We will test the effect of the CIP training (versus control group) on children´s social-emotional and linguistic development using linear regression analyses, adjusting for baseline levels of children´s social-emotional and linguistic development, respectively.

### Preliminary checks

Trials like the current one, especially with data collection taking place over an extended period of time (around 5 months for each data collection cycle, see Fig. 1), are liable to drop-out of participants. We will evaluate the randomness (vs. systematicness) of missing data. In order to maintain the original random allocation of (CPs and children within) care groups and counter potential biases in analyzing training effects, we will use an intent-to-treat approach in our analyses, by which the data of participants (CPs and children) who dropped out are analyzed as if they had completed the study, for instance through multiple imputation of missing outcome data [60].

Adjusting for covariates that help explain some of the variation in outcomes between participants can increase statistical power in randomized trials, yet is not commonly done [61]. Indeed, interventions trials in childcare similar to this one often do not report including covariates, apart from baseline levels of the outcome measure. It is therefore difficult to determine based on previous literature what covariates might enhance precision of estimation. We will evaluate the relevance of potential covariates to determine whether they should be included in the main analyses, including whether CPs did their training individually or in pairs, the caregiver-child ratio (number of caregivers per child) in the care groups during the video recordings, CPs´ educational background, and whether CPs in the control group had participated in any alternative training during the data collection period. For analyses of training effects on child development, we will test children´s age and the hours they spend in childcare per week as potentially relevant covariates. We will check if they (a) differ between the training and control group, and (b) are associated with the outcome. Covariates will be retained if they correlate ≥ 0.70 with the outcome, independently of whether they differ between the training and control group; they will be eliminated if they correlate ≤ 0.30 with the outcome; if they correlate 0.31-0.69 with the outcome, they will be retained if they are imbalanced between the training and control group [62]. For transparent publication and future meta-analytic purposes, we will report both unadjusted and adjusted models. In the case of non-significant findings, we will calculate Bayes Factors to indicate whether the null hypothesis or the alternative hypothesis predicts the data better, or whether the data cannot distinguish clearly between the null and alternative hypothesis [63, 64]. Before data analysis starts, all analyses will be pre-registered on the Open Science Forum (see https://osf.io/5fcs2/).

## Discussion

This trial evaluates whether the CIP training, which was found effective in improving the CPs’ interactive skills in a Dutch context [9], is effective in the Danish childcare setting. We expand on the original Dutch trial by also looking at potential positive effects on children’s social-emotional and linguistic development. If indeed the training leads to a childcare environment in which children are met with more sensitivity, respect for their initiatives and perspectives, a clearer structure, and more positive interactions with peers, then we expect this, based on developmental theory and research, to boost their social-emotional development. We expect that more frequent and high-quality verbal communication will affect children´s language development positively.

Many intervention trials in childcare are underpowered [15], because detecting the aggregate moderate (for caregivers) or small (for children) intervention effects requires very large samples which is generally extremely time consuming and labor intensive due to delivering of the interventions and coding observational measures. Our randomized trial aims to have a larger sample and therefore more statistical power than the studies included in the meta-analysis by Werner and colleagues [15]. This makes it feasible for us to detect the effects found by Werner and colleagues ( [15] p.268). Nevertheless there are constraints to the type of analysis we can do while retaining sufficient statistical power. We may therefore not be able to detect factors that affect the CIP training’s success in improving interactive skills in CPs, and in boosting children’s development.

Because we rely on childcare centers’ voluntary participation, we risk that the childcare centers enrolling in the study could do so, for example, because they generally pay attention to and work with child-CP interactions, and may have relatively high process quality already. This means that our sample may not be fully representative, in terms of baseline process quality, of the wider population of childcare centers in Copenhagen and Denmark. Therefore we would not necessarily be able to draw conclusions, based on our findings, applicable to the wider Danish childcare center population. In order to counter this, we keep track of childcare centers’ quality evaluations (conducted by the municipality) to make sure we don’t over-recruit childcare centers with evaluations of high quality, and we can steer recruitment toward centers with less positive evaluations as needed. We do the same for centers’ ESCS index, aiming to recruit centers with children of different ESCS backgrounds, as representative as possible of the Copenhagen population.

Additionally, because we recruit at the level of childcare groups, it is possible that participants allocated to the waiting-list control group benefit from the CIP training before taking part in it, either because (despite our request not to do so) participants in the CIP training group discuss training content with their colleagues, or because they show improved interactive skills and their behavioral change is observed by their colleagues in the waiting-list control group. When recruiting and at the start of each CIP training, we stress the importance of not discussing training content with colleagues in the control group until data collection is over.

Sensitive and stimulating interactions are crucial for the development of all children in child care, and can have a protective effect for children who may experience more adversity at home [7, 65]. Because interactions between CPs and children in more than a third of Danish nurseries have been found of insufficient quality in recent research, identifying effective interventions that can improve CP´s interactive skills is a priority in Denmark. If we found that the CIP training can be effective in the Danish context, this would be an important first step toward implementing training in these six interactive skills in Danish childcare. Additional research to evaluate whether the CIP training is effective for interactions with older children (4–5 years) would be necessary. Should the CIP training prove effective, we will evaluate the feasibility of integrating the CIP training in the Professional Bachelor Program in Social Education (which educates future CPs) at University College Copenhagen. In our view, it is of vital importance that future CPs get targeted behavioral training in interacting with infants and young children in a sensitive and stimulating way.

### Dissemination

We will communicate the findings of our study to the scientific community through publication in international, peer-reviewed journals and through presentations at international conferences. We will hold a conference for our study participants to discuss our findings with them. We will also communicate our findings to the wider public through publications in popular outlets.

## Data Availability

All participant data are stored electronically on a secured drive at the University of Copenhagen. After the study has been completed, data will remain stored in pseudonymized form on the same secured drive for five years. After that, data will be fully anonymized, and participant-level data may be made available upon reasonable request to external researchers, with prior ethics approval, on the basis of a clear research methodology employing the data. Any such data sharing will remain at the discretion of the Sponsor and Principal Investigator.

## Abbreviations

CIP: Caregiver Interactive Profile
CP: Childcare provider
